# Seeing the World as it is: Mimicking Veridical Motion Perception in Schizophrenia Using Non-invasive Brain Stimulation in Healthy Participants

**DOI:** 10.1007/s10548-018-0639-6

**Published:** 2018-03-07

**Authors:** Gorana Pobric, Johan Hulleman, Michal Lavidor, Gail Silipo, Stephanie Rohrig, Elisa Dias, Daniel C. Javitt

**Affiliations:** 1 Neuroscience and Aphasia Research Unit, Division of Neuroscience and Experimental Psychology, University of Manchester, Oxford Road, Manchester, M13 9PL UK; 2 Department of Psychology, Bar Ilan University, Ramat Gan, Tel Aviv, Israel; 3 Schizophrenia Research Division, Nathan Kline Institute, Orangeburg, NY 10962 USA; 4 Division of Experimental Therapeutics, Department of Psychiatry, Columbia University Medical Center, New York, NY 10032 USA

**Keywords:** Transcranial electrical stimulation, Motion perception, MT+, Schizophrenia

## Abstract

Schizophrenia (Sz) is a mental health disorder characterized by severe cognitive, emotional, social, and perceptual deficits. Visual deficits are found in tasks relying on the magnocellular/dorsal stream. In our first experiment we established deficits in global motion processing in Sz patients compared to healthy controls. We used a novel task in which background optic flow produces a distortion of the apparent trajectory of a moving stimulus, leading control participants to provide biased estimates of the true motion trajectory under conditions of global stimulation. Sz patients were significantly less affected by the global background motion, and reported trajectories that were more veridically accurate than those of controls. In order to study the mechanism of this effect, we performed a second experiment where we applied transcranial electrical stimulation over area MT+ to selectively modify global motion processing of optic flow displays in healthy participants. Cathodal and high frequency random noise stimulation had opposite effects on trajectory perception in optic flow. The brain stimulation over a control site and in a control task revealed that the effect of stimulation was specific for global motion processing in area MT+. These findings both support prior studies of impaired early visual processing in Sz and provide novel approaches for measurement and manipulation of the underlying circuits.

**Electronic supplementary material:**

The online version of this article (10.1007/s10548-018-0639-6) contains supplementary material, which is available to authorized users.

## Introduction

Though much work has been directed toward understanding higher-order cognitive and emotional impairments in schizophrenia (Sz), recent data have shown that lower-order sensory processing can also be affected (Butler and Javitt 2005; Javitt 2009; Chen et al. 2003; Martinez et al. 2012). Visual deficits in Sz are related to processing deficits in the magnocellular/dorsal stream, a pathway from the retina to the visual cortex and beyond. It is linked to visual motion processing and conveying signals related to low spatial frequencies, low contrast, and high temporal frequencies (Butler et al. 2001). These relative deficits in magnocellular function have been confirmed employing various methods, including steady-state visual event-related potentials (Martinez et al. 2012; Chen 2011) and fMRI using both static and motion stimuli (Haenschel et al. 2007; Martínez et al. 2008). The visual deficits are of potential clinical interest as they may provide clues to the underlying pathophysiology of cognitive dysfunction. Several studies report significant correlations between visual deficits and cognitive and social impairments in Sz (e.g. Chen 2011; Yoon et al. 2008). Deficits in early visual processing, moreover, significantly contribute to higher-level cognitive impairments such as recognition of emotional facial expressions, and social cognition (Martinez et al. 2007). Similarly, deficits in detecting coherent motion, contribute to deficits in recognition of biological motion (Kim et al. 2013) and theory of mind (Kelemen et al. 2005).

In this study, we investigated visual processing of motion in Sz patients and healthy controls. In Experiment 1 we tested Sz patients and healthy controls on a novel task in which a background optic flow field produces a distortion of the apparent trajectory of a moving stimulus, leading control participants to provide biased estimates of the true trajectory of motion under conditions of global stimulation (Warren and Rushton 2009). The aim of Experiment 2 was to understand the circuitry underpinning the performance of Sz patients in Experiment 1, by using non-invasive brain stimulation in healthy participants and exactly the same behavioural task.

**Fig. 1:**
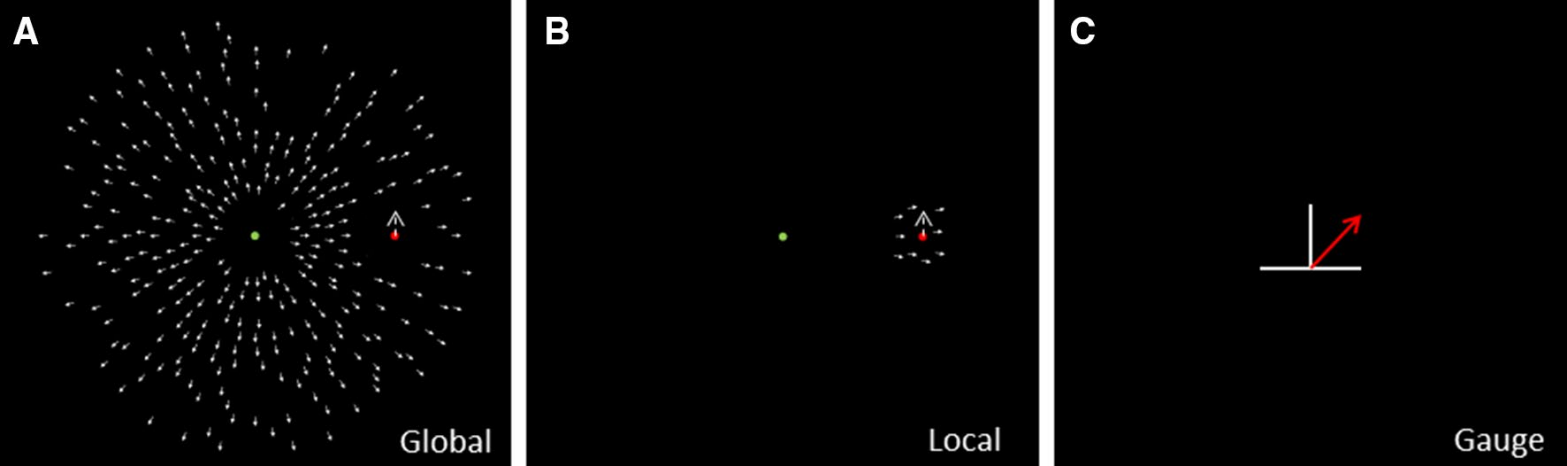
Stimuli. **a** Global task. Participants observed a radial optic flow field and a single upward moving red dot (probe) inside 1.3° radius aperture within the global optic flow field. Their task was to judge the perceived trajectory of an upward moving probe in a radial flow field. **b** Local Task. Inside a small 1.3° radius aperture participants observed a radial optic flow field and an upward moving red probe. **c** Response Gauge. Participants were indicating perceived direction of the probe by positioning the arrow gauge. The discrepancy between the onscreen probe movement and the perceived probe movement was used to determine the relative tilt

Optic flow fields are patterns of visual motion that the observer encounters while moving through the environment. Embedded in optic flow fields is information regarding self-motion and the structure of the environment (Gibson 1950; Koenderink 1986). When stationary observers judge the perceived trajectory of an upward moving probe object in a radial flow field, the probe is indeed perceived to be moving upward (matching the physical on-screen movement), but also toward the centre of the display (not present in the physical on-screen movement). This perceived motion to the centre is known as the relative tilt effect (Warren and Rushton 2009) (see Fig. 1a). We used the relative tilt effect as our index of motion processing. On average, healthy participants show a relative tilt effect of 30° when judging the on-screen trajectory of a moving probe (Warren and Rushton 2009).

Because processing optic flow and making judgements about relative tilt relies on the medial temporal complex MT+ (including the medial temporal (MT) and medial superior temporal (MST) areas) which receives prominent magnocellular system input (Morrone et al. 2000), we expected to see clear behavioural differences between healthy participants and Sz-patients. In healthy participants, “global” (low spatial-frequency) information is processed preferentially to “local” (high spatial-frequency) information (Sergent 1982). Therefore, in controls, the perceived tilt should be larger for global (large) flow fields than for local (small) flow fields.

However, in some tasks individuals with Sz perform paradoxically better than controls when processing local information in the presence of competing global information. For example, Place and Gilmore (1980) showed paradoxically superior numerosity discrimination in Sz patients in the presence of competing configural information. In addition, patients may show selectively impaired global vs. local motion processing (Chen et al. 2004). We therefore hypothesized that in Sz, the difference between global and local effects would be diminished. Because the flow field produces an illusory distortion of the motion trajectory, the predicted “deficit” in Sz is in the direction of a more veridical report of stimulus motion.

An advantage of this task is that impaired performance cannot be attributed to general factors such as inattention or lack of motivation by patients. Because foreground and background stimuli are superimposed, lack of attention would be expected to lead to increased, rather than decreased, susceptibility to the illusion. Furthermore, the global and local versions of the task are formally similar, so differential deficits must be attributed specifically to differential processing of the background information.

In addition to performance on the novel tilt task in Experiment 1, we also investigated global motion processing by determining coherence thresholds to translational and rotational motion in random-dot kinematograms (Newsome and Pare 1988; Chen et al. 2004). For both types of motion, the target for detection of coherent motion was a random dot pattern, displayed on a computer screen. The signal component was an array of dots moving coherently in one direction: (1) left or right—translational motion; (2) clockwise and anticlockwise—rotational motion. The noise component was another array of dots moving in random directions (Newsome and Pare 1988). The task employed random dot kinematograms (RDKs) at six motion coherence levels as stimuli. We predicted that “abnormal” (i.e. different from controls) but veridically more accurate performance on the relative tilt task in Sz would correlate with impaired motion processing detected using RDKs.

To study the mechanisms underlying the relative tilt effect, as well as potential future approaches to intervention for Sz deficits, Experiment 2 combined transcranial electrical current stimulation (TES), a non-invasive brain stimulation technique, with our optic flow task in healthy participants. TES uses low level (1–2 mA) currents applied via scalp electrodes to specific brain regions. It modulates neural activity in the stimulated regions in a polarity-dependent way. Studies of the motor cortex suggest that anodal stimulation enhances excitability of the underlying cortical areas and cathodal stimulation decreases it (Nitsche et al. 2008). Yet, in other perceptual and cognitive domains the polarity effects are much less clear (Jacobson et al. 2012; Fertonani et al. 2011). For example, Antal and colleagues (2004) studied motion coherence thresholds using TES over visual cortex. They reported that cathodal stimulation decreased the percentage of coherently moving dots necessary to detect the correct motion direction, suggesting improved motion perception (Antal et al. 2004).

Another way to modulate cortical processing is via transcranial random noise stimulation (tRNS). During tRNS a current of random intensity is delivered, with frequencies distributed across a specific range 0.1 and 640 Hz at a sampling rate of 1280 samples per second with no overall direct current offset. tRNS is divided into low-frequency LF-RNS (frequencies from 0.1 to 100 Hz) and high frequency random noise stimulation HF-RNS (frequency range from 101 to 640 Hz). This frequency spectrum looks similar to the “white noise” characteristic. Terney et al. (2008) reported tRNS has a consistent excitability increase lasting at least 60 min, both on physiological and behavioural measures. HF-RNS applied concurrently with a cognitive or motor task has been shown to improve performance, presumably by increasing cortical excitability (Prichard et al. 2014). We expected TES of MT+ to interfere with the processing of optic flow in the global condition, but not in the local condition. Based on prior TES studies of motion coherence (Antal et al. 2006, 2012), we hypothesized that cathodal TES would increase the perceived trajectory bias. To the extent that such an effect could be produced, it would suggest the potential utility of TES in future studies of motion processing remediation in Sz.

## Materials and Methods

### Experiment 1

#### Participants

Patients—16 Sz (4 females, mean age = 43, SD = 10.9) took part in the experiment. Sz patients were tested at the Nathan Kline Institute for Psychiatric Research (NKI) in Orangeburg, NY. 13 Sz participants met DSM-IV (SCID-defined) criteria for schizophrenia and 3 patients met DSM-IV (SCID-defined) criteria for schizoaffective disorder (American Psychiatric Association 1994). 5 Sz patients were inpatients at Rockland Psychiatric Center (Orangeburg, NY) the other 11 patients were recruited from outpatient clinics in southern New York State and northern New Jersey.

All patients were clinically stable and on a stable dose of second generation antipsychotic medication at the time of testing. All participants had normal or corrected-to-normal visual acuity (20/40 or better monocular and 20/25 or better binocular) as assessed with the Logarithmic Visual Acuity Chart (Precision Vision). Symptom severity was measured with the Positive and Negative Syndrome Scale (PANSS) (Kay et al. 1987). PANSS assesses positive symptoms, negative symptoms, and general psychopathology on three scales. Cognitive functioning in patients was assessed using subtests from the MATRICS Consensus Cognitive Battery (MCCB) for the following domains: speed of processing, attention/vigilance, working memory, visual learning, and reasoning/problem solving (Kern et al. 2011). For more background testing information on Sz patients please see Table 1. A detailed descriptions of tests can be found in Online Appendix.

**Table 1:** Demographic information for schizophrenia patients

	Mean	SD
Age (years)	43.1	9.6
Quick IQ	97.4	7.1
SES	25.75	8.6
Illness duration (years)	8.9	8.9
CPZ equivalent (mg)	685	459
Education (years)	11.8	2.1
PANSS Positive Scale	20.26	5.2
PANSS Negative Scale	18.67	3.8
PANSS General Psychopathology Scale	37.4	7.6
WAIS IV Block Design	41.63	11.5
WAIS IV Visual Puzzles	12.64	4.6
WAIS IV Matrix Reasoning	11.36	4.1

*SES* Socioeconomic status Scale, *CPZ* chlorpromazine, *PANSS* Positive and Negative Syndrome Scale, *WAIS* Wechsler Adult Intelligence Scale

#### Control Participants

15 (7 females, mean age = 41, SD = 6.17) age-matched healthy controls took part in testing. They were recruited and tested at the University of Manchester, UK.

All study procedures were approved by the local ethics committees (NKI, US and UREC, UK) and conducted in accordance with the Declaration of Helsinki. Written informed consent was obtained from all observers (patients and controls) after full explanation of procedures.

## Tilt Task

### Apparatus and Stimuli

At NKI, the visual stimuli were displayed on a 22″ CRT monitor IIyama (Vision Master Pro 450, Iiyama North America) controlled by ATI FireGL v3400 graphics card. For the testing age-matched controls at the University of Manchester UK, the stimuli were displayed on ViewSonic vx2268wm, 22″ CRT monitor controlled by a NVidia GeForce gt440 graphics card. In both labs the CRT monitors had the same frame rate of 100 Hz and were positioned 57.3 cm from the participant. The optic flow field was generated as a virtual cloud of 300 dots in a 3D volume. The onscreen dot location was first sampled from a uniform 2D distribution (to keep average dot density constant over the display). The 3D location of each dot in the scene was then randomly sampled in a simulated depth range between 0.5 and 1.5 m from the observer. The dots were presented in a circular aperture with radius of approximately 15 deg visual angle. The dot motion was appropriate for an observer moving forwards at a speed of around 0.59 m/s. Dot density was maintained over the course of the stimulus presentation by re-positioning any dots which moved off the screen or had been present in the display for longer than 20 frames (200 ms). The red probe dot moved within an aperture of 1.5° radius, 4° away from the centre (to the left or right). In order to control for anticipatory responses of the onscreen probe trajectory, the probe moved along an upward trajectory with angle 75°, 90° or 105°. The background flow field was either global (flow field across the screen, except the aperture containing the probe) and local (flow field only within the aperture with the probe).

#### Procedure

First, participants were shown the response gauge (Fig. 1c—an arrow that could be rotated either clockwise or counter-clockwise) and given the opportunity to undertake some practice trials. Each trial consisted of a 2 s presentation of the moving probe and the flow field (either global or local). Throughout, the participant was instructed to maintain fixation on a small circular dot at the centre of the display. After the motion presentation the participant saw the response gauge and their task was to set the adjustable paddle gauge (superimposed line) to match the trajectory of the probe during the motion presentation by moving the mouse and pressing the left or right mouse key (task: please indicate the direction of the moving probe). Participants were presented with two experimental flowfield conditions: global and local (See Fig. 1a, b). We recorded their actual response in degrees. Participants saw eight repetitions of each of the conditions over a single experimental session of around 20 min. A video clip od the task can be found in Supplementary Methods.

## Random Dot Motion Task

### Apparatus and Stimuli

Stimuli were presented on a CRT 20″ monitor (Mitsubishi RDF223H) controlled by a CRS ViSaGe graphics card. The CRT had a frame rate of 60 Hz and a pixel resolution of 0.04°/pixel. The task employed random dot kinematograms (RDKs) containing 100 dots at six motion coherence levels: 0, 5, 10, 20, 40, and 100%. The RDKs were 23° wide and 17° high. Each RDK was composed of individual “dots” (1.75 by 2.25 mm) with a luminance of 70 cd/m^2^. The dots were randomly distributed with a density of 29% on a 0.013 cd/m^2^ background. The luminance contrast between dots and background was calculated as 99.9% (Michelson contrast ratio). Dots were put into motion for 1.5 s (90 frames at a rate of 0.0167 s per frame). The direction of global dot motion defined the display: i.e., a proportion of the dots drifted coherently in one direction (left or right/clockwise or anti-clockwise) while the remaining dots moved in random directions. Dot speed was 4.8°/s. Sz patients perceived these displays as surfaces drifting to the right or to the left when judging translational motion or clockwise or anti-clockwise when judging rotational motion. The stimulus strength was varied by changing dot coherence (the proportion of dots drifting in a single direction). Visual stimuli were generated using MATLAB 64 bit version R2013a (The MathWorks, Natick, MA, USA) with Psychophysics Toolbox-3 extensions.

#### Procedure

Sz patients sat approximately 60 cm from the computer screen. Their task was to judge the direction of global motion (left or right or clockwise–anticlockwise) for each stimulus presented in a two-alternative forced-choice paradigm. The participants would either respond “left” or “right”, “clockwise” or “anti-clockwise” by pressing two designated computer keys.

During a session, participants were given 60 practice trials in total. For translational motion, Sz patients completed 30 practice trials (10 trials each at 100, 20, and 5% coherence). They did the same for rotational motion. This was followed by 120 test trials, blocked by motion (60 translational and 60 rotational motion trials) and counterbalanced across participants. In each block, the stimuli were randomly selected from trial to trial. A Weibull function was fit to the data from each block (translation and rotation) for each subject. Motion coherence thresholds were defined as the percentage of coherence corresponding to the 75% correct level of the Weibull fit.

## Experiment 2—Transcranial Electrical Stimulation

### Power Analysis

The relative tilt effect is a robust phenomenon (Warren and Rushotn 2009). The effect size in Experiment 1 for the comparison between Sz patients and controls in the global optic flow condition was 1.02. An a priori power analysis (G*Power version 3.1.3; Faul et al. 2007) indicated that a sample size of ten participants would be able to detect this effect size with a power of 0.80 at an alpha of 0.05 in a within-subjects design.

### Participants

12 participants (6 females, mean age 24, SD = 2.5) took part in Experiment 2a and 9 participants (7 females) participated (mean age 22.3, SD = 2.6) in Experiment 2b (control experiment). All participants were recruited at the University of Manchester. The study was approved by the local ethics committee and conducted according to the Declaration of Helsinki. Written informed consent was obtained from all participants after full explanation of procedures.

### Design

This was a within subject design in which participants were randomly assigned to a stimulation condition and the order of stimulation sessions was counterbalanced across participants. Each participant completed four sessions (separated by minimally 7 days), with a different type of stimulation (cathodal, anodal, HF-RNS and sham) administered in each. On each day they saw both global and local flow fields. This design was the same for both Experiments 2a and b that differed only in the critical stimulation location: right MT+ in Experiment 2a, and right ATL in Experiment 2b. Right ATL stimulation was included to control for site specific effects following stimulation of MT+ region.

Apparatus, stimuli and procedure—the experiment was carried out on exactly the same apparatus, with same stimuli and procedure as our behavioural tilt task (see Exp. 1—age-matched controls, University of Manchester).

#### TES

**Fig. 2:**
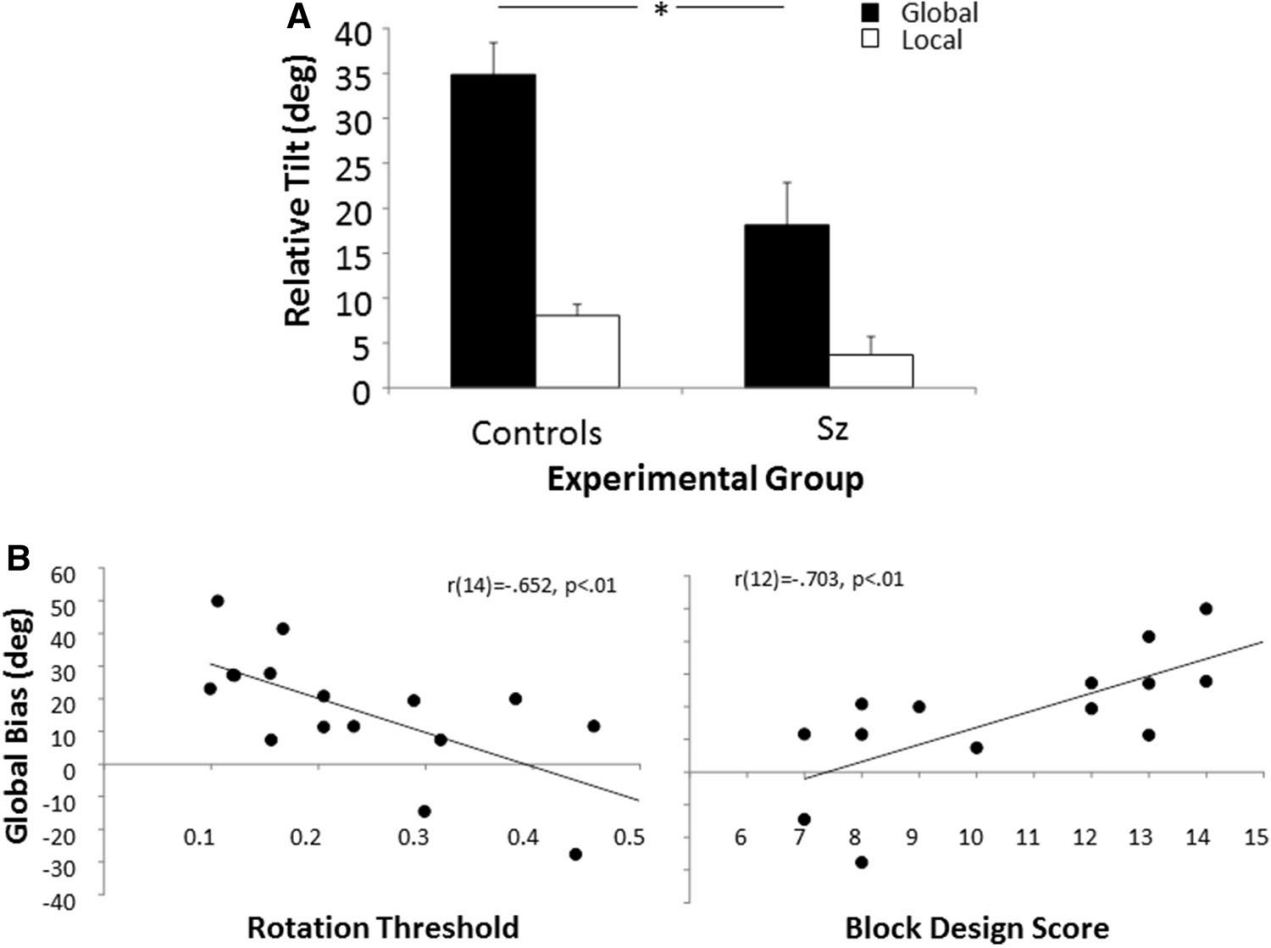
Behavioural results. **a** The relative tilt for global and local flow fields in control participants and schizophrenic patients (Sz). Sz patients show significantly reduced global tilt compared to control participants. Error bars indicate standard error of mean (SEM). **b** Correlations between global task performance in Sz patients and (left panel) perceptual rotation threshold (proportion coherent dots) and (right panel) score on Block Design test

Direct current was generated by a NeuroConn stimulator (Rogue Resolutions) and delivered via a pair of differently sized electrodes, a stimulating square scalp electrode (5 × 5 cm) and a reference electrode (5 × 7 cm), covered with conductive rubber and saline-soaked synthetic sponges. The active electrode was placed approximately 3–4 cm above the mastoid-inion line and 6–7 cm right of the midline in the sagittal plane (right MT: Fig. 2a). In Experiment 2b the right ATL position corresponded to FT8 electrode position. The electrode positions were selected on the basis of previous imaging and TMS studies of MT+ (Walsh et al. 1998) and right ATL (Pobric et al. 2016). The reference electrode was placed over Cz. The current was ramped-up and down for 15 s. tDCS (anodal and cathodal) was applied concurrently with the task for 20 min with an intensity of 1.5 mA. For HF-RNS, the current was delivered in the form of high frequency noise (101–640 Hz). The current intensity was 1.5 mA peak-to-peak, with each sample being drawn from a normal distribution with mean 0 µA, and with 99% of all generated amplitude values lying between − 750 and + 750 µA. Stimulation (15 s ramp-up and 15 s ramp-down) always started at the same time as the onset of the motion task. For sham stimulation, the stimulator was turned on for 30 s (15 s ramp-up and 15 s ramp-down) after which it was switched off. The sham condition produces the sensation of being stimulated, but does not induce neurophysiological changes that can influence performance (Ambrus et al. 2011).

## Results

### Behavioural Results—Experiment 1

An exploratory data analysis was conducted to determine if the size of relative tilt performance was normally distributed. Results for the Kolmogorov–Smirnov test found no deviation from normality for the patient group D(16) = 0.173, p > .05 nor the control group, D(15) = 0.151, p > .05.

The difference between onscreen and perceived trajectories was submitted to a mixed ANOVA with flow field type (global, local) as within and experimental group (Sz patients vs. controls) as between factor. A main effect of flow field was observed [Wilks’ Lambda = 3.25, F(1, 29) = 60.16, p < .001], as well as a significant interaction between flow field and experimental group [Wilks’ Lambda = 0.844, F(1, 29) = 5.374, p = .028]. Planned t-tests compared the performance of Sz patients and matched controls for each flow field. There was a significant difference in processing motion trajectories for global flow fields only [t(29) = 2.82, p = .009]. Sz patients showed a significantly reduced bias in trajectory perception for global motion processing (See Fig. 2a).

Additionally, a Pearson product-moment correlation coefficient was computed to assess the relationship between Sz patients’ relative tilt score and their performance on motion coherence thresholds and neuropsychological tests of visuospatial processing. Tests of this type are specifically sensitive to deficits within dorsal stream, in particular the MT+ region (Bisley and Pasternak 2000). As predicted less illusory bias on the global (i.e. more veridically accurate performance) task, correlated significantly [r(14) = − .652, p < .01] with impaired motion detection ability on the rotational motion task. Correlation between the global task and translational motion performance was not significant [r(14) = 0.149, p = .60]. Performance on the global task also correlated with the block design subtest of the WAIS-IV tests (general background testing for Sz patients) [r(12) = − .703, p < .01] (Wechsler 1981) (See Fig. 2b).

## TES Results

One participant did not complete the Experiment 2 and was excluded from the analyses.

### Experiment 2a: MT+

**Fig. 3:**
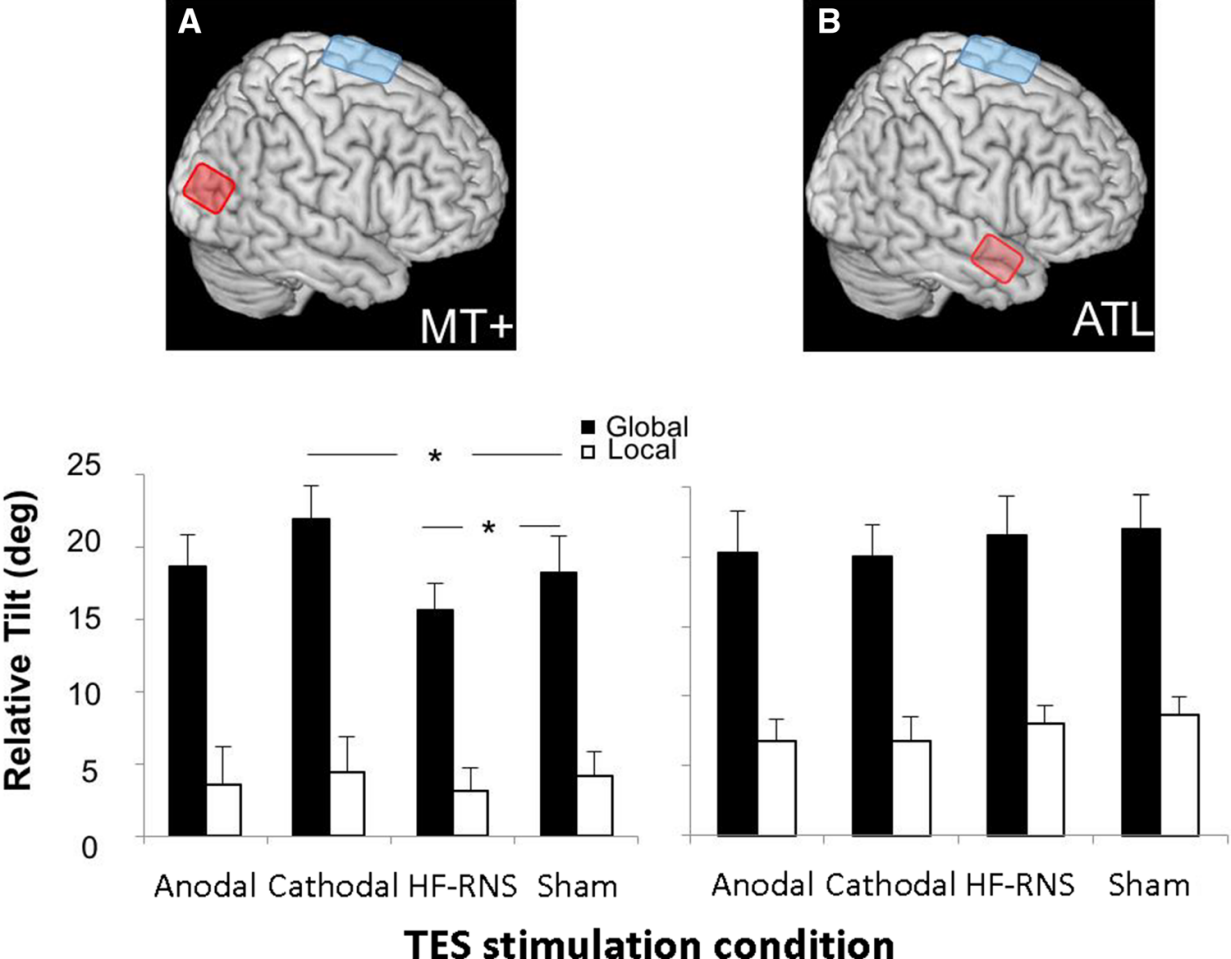
TES results. **a** MT+ region and TES electrode montage. Anodal and cathodal refer to tDCS stimulation. HF-RNS represent high frequency random-noise stimulation. **b** ATL region and TES electrode montage. Anodal and cathodal refer to tDCS stimulation. HF-RNS represent high frequency random-noise stimulation. Each bar represents the TES modulation of relative tilt effect for global and local optic flow fields. Error bars indicate standard error of mean (SEM) adjusted to reflect the between-condition variance used in repeated-measure designs (Loftus and Mason 1994). *MT* cortical middle temporal area, *ATL* anterior temporal lobe, *TES* transcranial electrical stimulation, *HF-RNS* high frequency random noise stimulation

The difference between onscreen and perceived trajectories for all participants and all conditions were submitted to a repeated-measures ANOVA with two within-subjects factors: stimulation (cathodal, anodal, HF-RNS and sham), and optic flow field (global, local). A main effect of stimulation approached significance [Wilks’ Lambda = 0.457, F(3, 8) = .897, p = .086], a main effect of flow field was observed [Wilks’ Lambda = 0.246, F(1, 10) = 30.717, p < .001], as well as a significant interaction between the two was observed [Wilks’ Lambda = 0.241, F(3, 8) = 8.390, p = .007]. Planned t-tests were used to compare performance for each flow field (global and local) and stimulation type. After controlling for the False Discovery Rate (FDR) (Benjamini and Hochberg 1995), we found that cathodal stimulation significantly increased the relative tilt bias [t(10) = 2.64, p < .05], while HF-RNS significantly decreased it [t(10) = 2.65, p < .05] (see Fig. 3a), producing a more veridical performance.

### Experiment 2b: rATL

The difference between onscreen and perceived trajectories for all participants and all conditions were submitted to a repeated-measures ANOVA with two within-subjects factors: stimulation (cathodal, anodal, HF-RNS and sham), and optic flow field (global, local). Only a main effect of flowfield was observed [Wilks’ Lambda = 0.141, F(1, 8) = 48.872, p < .001]. There was no significant main effect of stimulation type [Wilks’ Lambda = 0.690, F(3, 6) = .897, p = .495] or interaction [Wilks’ Lambda = 0.837, F(3, 6) = .390, p = .765] (see Fig. 3b).

Additionally, we directly compared the cathodal and HF-RNS stimulation effect in the MT + and rATL regions. We computed the difference between cathodal and sham stimulation and between HF-RNS and sham stimulation for both regions and submitted these differences to independent t-test analyses. We found a significant stimulation effect over the MT+ region compared to the rATL region for cathodal stimulation [t(18) = 2.115, p < .05] and HF-RNS stimulation [t(18) = − 2.429, p < .05].

**Fig. 4:**
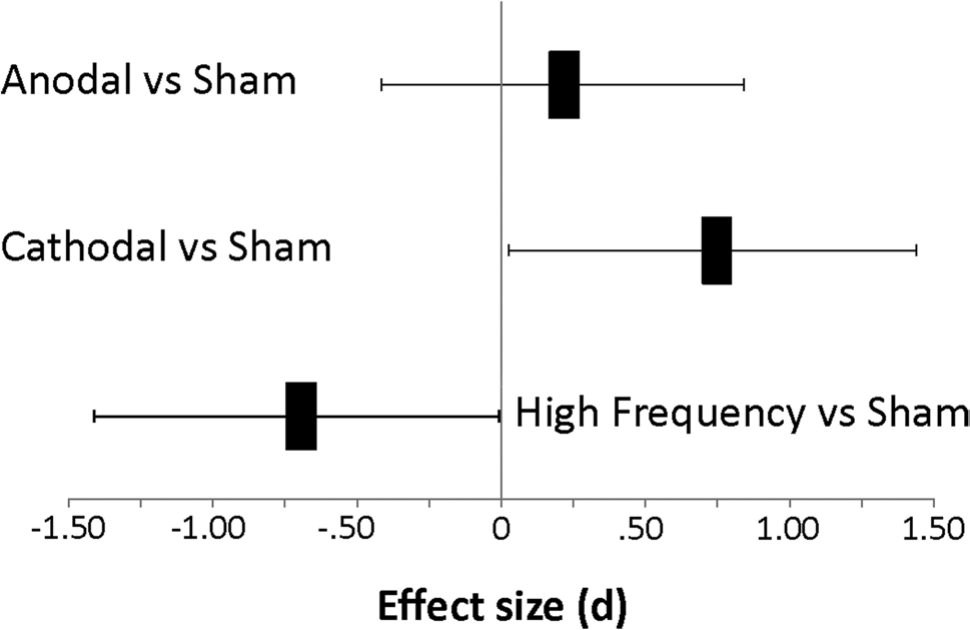
Effect sizes of global relative tilt following TES stimulation

To further compare the TES results of MT+ stimulation, effect sizes for relative tilt were calculated based on the difference in performance between the sham and stimulation session using Cohen’s d (Cohen 1992). Figure 4 illustrates the effect sizes for relative tilt from each condition relative to sham. The effect-size associated with anodal stimulation was small (d = 0.1), whereas cathodal (d = 0.84) and HF-RNS (d = − 0.79) produced opposite direction, large size effects. (See Fig. 4).

**Fig. 5:**
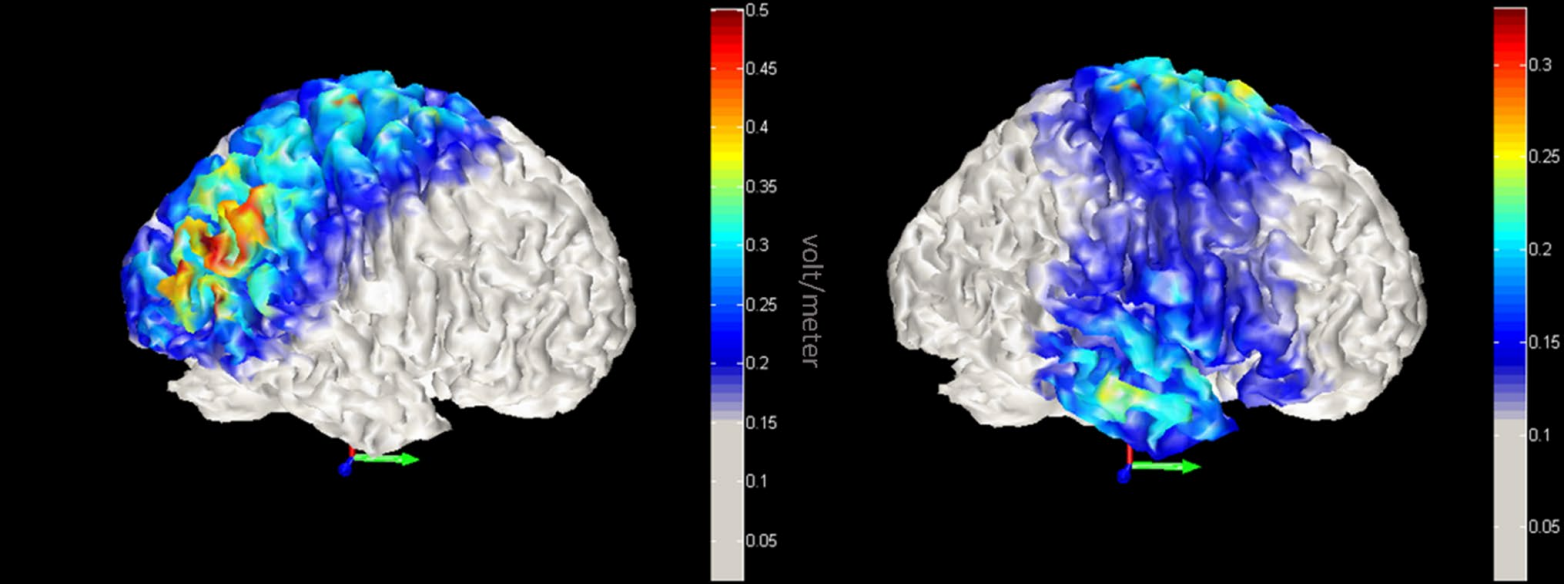
Model of transcranial direct current stimulation (tDCS) current. Red-yellow colours indicate increased magnitude of the total electric field due to tDCS. Left panel displays right MT+ stimulation, while the right panel highlights stimulation within the right anterior temporal lobes

In order to confirm topographical effects of neuromodulation, we modelled the magnitude of the total electric field due to stimulation with COMETS (Jung et al. 2013). The model provided evidence that the tDCS electric field was largest over the right MT+ region and right ATL (See Fig. 5).

## Discussion

In this study, we used a behavioural task that assessed the local and global contributions to trajectory perception of a moving probe in an optic flow field. We found a significantly reduced bias in trajectory perception for global motion processing in Sz patients compared to healthy controls. This shows that Sz patients were less affected by the global background motion, as indexed by the reduced relative tilt effect. As all participants were instructed to provide their best estimate of trajectory irrespective of background stimulation, the superior performance of Sz patients on this task can only be attributed to reduced sensitivity to background optic flow.

To our knowledge, our finding of paradoxically better performance (i.e. closer to veridical) during motion processing in schizophrenia in a tilt illusion task is novel. In addition, it is consistent with the classic findings of Place and Gilmore (1980) that Sz patients were less affected by the perceptual organization of the arrays in a numerosity task. Patients are also less affected than controls by a variety of visual illusions such as stereopsis, Ponzo illusion or Hermann grid, even though they are less affected by others (e.g. Muller-Lyer), potentially related to impaired contrast gain within the early visual system (Kantrowitz et al. 2009).

The visuospatial abilities of the Sz patients, as assessed by the block design (BD) subtest of the WAIS-R battery, correlated positively with the size of the relative tilt effect. This result is reminiscent of the reports of reduced susceptibility to visual illusions and good performance on the BD in autism (Spencer and O’Brien 2006; Happé 1996). There was also a negative correlation between the relative tilt effect and rotational motion coherence thresholds in the Sz patients. This suggests involvement of areas MT + and MST in the tilt effect, since previous studies have shown critical involvement of these areas in processing rotational random dot kinematograms (RDK) (Morrone et al. 1995, 2000).

To study the underlying neural mechanism of the relative tilt effect and the involvement of area MT+ in optic flow processing, we used TES in healthy participants. We found that cathodal and HF-RNS stimulation had opposite effects on trajectory perception in the global optic flow condition. While HF-RNS stimulation reduced the bias in trajectory perception, cathodal stimulation increased it. This pattern was specific to MT+ stimulation, and was not found when a control area (right ATL) was stimulated. The parallelism between the Sz patient data and these HF-RNS results in healthy participants is consistent with the notion that MT+ region is crucial for global motion processing. On the other hand, cathodal stimulation increased the bias.

There are several interpretations for the polarity effects observed in our TES experiments. In the global condition of our optic flow task, participants were processing two competing stimuli: the radial optic flow and the upward moving probe dot. In agreement with Antal et al. (2004), we suggest that cathodal stimulation decreased global neural activity. This resulted in inhibition of neural activations from the dominant global radial optic flow field but, at the same time, it also reduced the already weaker activations from the upward moving single dot, pushing them under the activation threshold. Consequently, cathodal stimulation amplified the global motion signal, increasing the relative tilt of the moving dot. In the local condition, the difference in neural activity between flow field and the moving dot is much smaller, therefore, cathodal stimulation affected both activations equally and performance did not change.

We also found that HF-RNS stimulation reduced relative tilt. The mechanism of action of HF-RNS might be based on repeated subthreshold stimulations, which may prevent homeostasis of the system and potentiate task-related neural activity (Fertonani et al. 2011; Pirulli et al. 2014). Evidence suggests that HF-RNS improves the detection of weak neuronal signals (Miniussi et al. 2010), which facilitates information processing in the brain (Stein et al. 2005; Li et al. 2006). This is analogues to the stochastic resonance phenomenon where a signal that is normally too weak can be boosted by adding white noise to the signal. The frequencies in the white noise corresponding to the original signal’s frequencies will resonate with each other, amplifying the original signal while not amplifying the rest of the white noise.

Recently, it has been demonstrated that adding the optimal level of random noise stimulation to visual cortex, has a signal enhancing-effect for weak stimuli and results in improved visual detection accuracy (van der Groen and Wenderoth 2016). Therefore, it is plausible that HF-RNS activated neurons are more sensitive to weaker inputs, amplifying the signal from the moving dot. This would result in a more veridical perception of a moving probe in an optic flow task.

It may seem surprising that we did not find an effect of anodal stimulation in our experiments, because anodal tDCS is thought to induce excitatory changes in the underlying brain tissue (Nitsche et al. 2008). Both anodal tDCS and HF-RNS over motor cortex resulted in motor-evoked potential (MEP) increases using the same stimulation parameters and electrode sizes (Moliadze et al. 2010).

But even in the motor cortex, differences between tDCS and HF-RNS have been observed. In a recent study (Moliadze et al. 2014) measured motor-evoked-potential amplitudes (MEPs) in a fixed time sequence following different TES protocols during stimulation of primary motor cortex (M1). Although both tRNS and anodal tDCS had excitatory effects on M1, HF-RNS stimulation produced the strongest MEPs, while anodal tDCS significantly increased MEP duration compared to sham stimulation.

More importantly, results obtained within the motor system are not always equivalent to the results obtained in the visual system or other cortical areas (Antal et al. 2004; Moliadze et al. 2005). For example, anodal stimulation applied over the primary visual cortex, had no effect on perceptual learning while HF-RNS improved it (Ferotnani et al. 2011). Zito et al. (2015) report significant improvement in motion perception in the left hemifield after cathodal HD-tDCS over right V5, but not in shape perception. Sham and anodal HD-tDCS did not affect performance on either task. Recently, Battaglini et al. (2017) have shown that both anodal and cathodal stimulation over MT+ region improve discriminability of the coherent motion detection, albeit through different mechanisms. While anodal stimulation reduced the threshold of motion coherence, cathodal stimulation reduced the steepness of the slope, indicating noise reduction.

Previously it has been reported that external stimulation of MT+ by TMS leads to impaired motion direction discrimination (Laycock et al. 2007; Tadin et al. 2011) supporting the dominant role of MT+ in coding of motion (Morrone et al. 1995, 2000; Antal et al. 2004). However, we found that the application of inhibitory, cathodal stimulation does not impair motion direction discrimination as might be expected based on a direct analogy between inhibitory TMS paradigms and cathodal stimulation (Nitsche et al. 2008). Thus, it seems that TMS and cathodal stimulation over area MT+ have opposing effects on motion perception. This adds to a growing body of evidence suggesting that cathodal stimulation can have facilitating effects in visual and cognitive domains (Antal et al. 2004; Tadin et al. 2011; Dockery et al. 2009; Weiss and Lavidor 2012; Filmer et al. 2015). We report high specificity of brain stimulation over area MT+, since stimulation applied over the ATL, a brain area that is not involved in processing optic flow, was ineffective. Using HF-RNS stimulation over area MT+, we showed that the behavioural pattern seen in patients with schizophrenia (Kim et al. 2006; Tadin et al. 2006) can be mirrored in neurologically intact participants. Specifically, we demonstrated that temporary interference with neural processing in area MT+ produces a selective impairment of global motion processing.

While we demonstrate task and site specific modulation of trajectory perception, some caution is needed when interpreting mechanisms that drive this effect. It is generally assumed that the region most affected by stimulation is right under the electrodes, however some studies suggest that maximal current flow occurs between the electrodes (Datta et al. 2012). Here, we explicitly modelled the current flow and showed that cortical current density distributions were highest over the MT+ region in Experiment 2a and right ATL region in Experiment 2B. In translational studies with clinical populations, extra consideration should be taken when establishing baseline levels of cortical excitation that may influence the efficacy and direction of stimulation effects.

To conclude, in healthy volunteers, HF-RNS over MT+ reproduced the pattern of reduced sensitivity in observed in Sz patients, relative to both sham MT+ stimulation and HF-RNS over a control region (ATL). By contrast, cathodal stimulation increased sensitivity in healthy volunteers. These findings both support prior studies of impaired early visual processing in schizophrenia and provide novel approaches both for measurement and manipulation of the underlying circuits.

Finally, our results support the use of HF-RNS along with tDCS for local stimulation, and reaffirms the utility of TES for modulation of local brain function. Although visual sensory deficits in schizophrenia are now well documented using behavioural, neurophysiological and neuroimaging based approaches, in virtually all paradigms performance of schizophrenia patients is worse than that of controls, raising concerns that issues such as motivation and cooperation may contribute to between group differences. In the future, the stimulation effects observed in this study should be combined with perceptual training paradigms to improve motion perception in patients with magnocellular dysfunction.

## Electronic Supplementary material

Below is the link to the electronic supplementary material.

Supplementary material 1 (DOCX 18 KB)

Supplementary material 2 (WMV 3810 KB)
