# Supplementary material for: Seeing the World as it is: Mimicking Veridical Motion Perception in Schizophrenia Using Non-invasive Brain Stimulation in Healthy Participants
Source: Brain Topogr. 2018 Mar 7;31(5):827–37. doi: 10.1007/s10548-018-0639-6 (PMC6097741; doi:10.1007/s10548-018-0639-6)
Supplement: Supplementary file 1 — Supplementary material 1 (DOCX 18 KB) [file 10548_2018_639_MOESM1_ESM.docx]

*Appendix 1 - Description of tests used to evaluate patients with schizophrenia*

1. All patients were evaluated on the **MATRICS** Consensus Cognitive Battery (MCCB - <http://www.matricsinc.org/mccb/>). MCCB is intended to provide a relatively brief evaluation of key cognitive domains relevant to schizophrenia and related disorders. For this study, patients were tested on 6 cognitive domains (speed of processing, attention/vigilance, working memory, verbal learning, visual learning reasoning and problem solving).
2. The **Wechsler Adult Intelligence Scale** (**WAIS**) is an IQ test designed to measure intelligence and cognitive ability in adults and older adolescents (Mattarzzo, J. 1972). The current version, WAIS-IV is composed of 10 core subtests and five supplemental subtests, with the 10 core subtests comprising the Full Scale IQ. We report Block Design is a test in the perceptual organization domain. The test-taker uses hand movements to rearrange blocks that have various color patterns on different sides to match a pattern (shown to them on cards). The items in a block design test can be scored both by accuracy in matching the pattern and by speed in completing each item. Proposed abilities measured: Spatial perception , visual abstract processing, and problem solving.
3. **The Quick test** is a nonverbal IQ test. The Quick test has 3 picture forms that contain 4 pictures each and each picture on the form is numbered 1-4 (I have attached a sample of one of the forms). For each of the forms, words (easy to more difficult) are read one at a time to the participant. The participant is instructed look at the pictures on the form and to point to or give the number of the picture that best describes the meaning of the word. All three of the forms can be administered, or one or 2 forms can be administered and norms (mental age, IQ and percentile) can be calculated based on number correct (Ammons & Ammons, 1962)
4. **SES (**Socioeconomic Status)

For patients SES is computed based on the participants education and occupation. We also computed parental SES for patients. Participant and parental occupation were coded using a nine-point scale and education were coded using a seven-point scale. The range for occupation was 1 (Farm laborers, menial service workers (dependent on welfare, no regular occupation) to 9 (higher executives, proprietors of large businesses, major professional) and the range for education was 1 (less than 7^th^ grade) to 7 (graduate/professional training (graduate degree)) (based on Hollingshead's 4-factor index of social status (see Hollingshead, 1975). For patient SES (participant SES), we then computed a Participant Index which is based on the formula ((Occupation score x 5) + (Education score x 3)) (see Hollingshead, 1975). If the participant has a spouse who is gainfully employed, both the participant and spouse index was computed and the average SES was calculated, otherwise the participant data alone was used. For parental SES, when both the occupation and education for the parent was unknown, the individual parent score was not computed. The combined parent score is computed using the same formula noted above. The mean of the mother and father occupation was taken when data for both parents were available (same as participant and spouse). When occupation and education data for only one parent was known, parent SES was computed using the single parent score.

References:

Ammons, R. B., & Ammons, C. H. (1962). The Quick Test (QT): Provisional manual. *Psychological Reports, 11*(1), 111-161.

Hollingshead, A. A. (1975). Four-factor index of social status. Unpublished manuscript, Yale University, New Haven, CT.

Matarazzo, Joseph D. (1972). *Wechsler's Measurement and Appraisal of Adult Intelligence* (5th and enlarged ed.). Baltimore (MD): Williams & Witkins

Nuechterlein KH, Green MF, Kern RS, Baade LE, Barch DM, Cohen JD, Essock S, Fenton WS, Frese FJ III, Gold JM, Goldberg T, Heaton RK, Keefe RSE, Kraemer H, Mesholam-Gately R, Seidman LJ, Stover E, Weinberger DR, Young AS, Zalcman S, Marder SR (2008) The MATRICS Consensus Cognitive Battery, part 1: test selection, reliability, and validity. Am J Psychiatry (doi: 10.1176/appi.ajp.2007.07010042)

Weiss, Lawrence G.; Saklofske, Donald H.; Coalson, Diane; Raiford, Susan, eds. (2010). *WAIS-IV Clinical Use and Interpretation: Scientist-Practitioner Perspectives*. Practical Resources for the Mental Health Professional. Alan S. Kaufman (Foreword). Amsterdam: Academic Press.
